# A study on prevention of bleeding complications using lusutrombopag for safe RFA in patients with hepatocellular carcinoma with low platelet counts: prospective observational study

**DOI:** 10.1186/s12876-023-02879-0

**Published:** 2023-07-24

**Authors:** Hideo Yoshida, Takamasa Ohki, Mineo Kanezaki, Takuma Teratani, Shinpei Sato, Shuntaro Obi, Takahisa Sato, Masatoshi Akamatsu, Koji Uchino, Hiroyoshi Taniguchi

**Affiliations:** 1 Dept. of Gastroenterology and Hepatology, Japanese Red Cross Medical Center, Tokyo, Japan; 2 Division of Gastroenterological Medicine, Mitsui Memorial Hospital, Tokyo, Japan; 3 Dept. of Gastroenterology, NTT Medical Center Tokyo, Tokyo, Japan; 4 Dept. of Gastroenterology and Hepatology, Kyoundo Hospital, Tokyo, Japan; 5 Dept of Gastroenterology, Teikyo University Chiba Medical Center, Ichihara, Chiba Japan; 6 Dept. of Gastroenterology, JR Tokyo General Hospital, Tokyo, Japan

**Keywords:** Hepatocellular carcinoma, Platelet count, Radiofrequency ablation, Locolegional therapy, Invasive procedure, Lusutrombopag, Hepatitis, Cirrhosis, Liver tumors, Thrombopoietin receptor

## Abstract

**Background:**

Platelet (PLT) transfusion was the most practical way to increase patients’ PLT counts before invasive hepatic procedures such as radiofrequency ablation (RFA) for hepatocellular carcinoma (HCC). A novel drug that raises the PLT count by acting on the thrombopoietin receptor has recently become available.

**Methods:**

Lusutrombopag 3 mg was administered daily for 7 days to patients who underwent RFA for liver tumors with low PLT counts (< 50,000 PLT µL^− 1^). We collected demographic data concerning the patients’ liver function and PLT counts.

**Results:**

Lusutrombopag was administered to 91 patients, with a median age of 71 years (range 51–86). Forty-two patients had hepatitis C, 12 had hepatitis B, 21 had alcoholic liver disease, 11 had nonalcoholic steatohepatitis, and five had other diseases. The median Child-Pugh score was 7 (range 5–11). Thirty-seven patients had stage I tumors, 41 had Stage II, 12 had stage III, and one had stage IV. PLT count was elevated from 4.4 × 10^4^ ± 1.4 × 10^4^ to 8.6 × 10^4^ ± 2.5 × 10^4^ PLT µL^− 1^. Lusutrombopag administration prevented PLT transfusions in 84/91 patients (92%). No patient had bleeding complications after RFA. One had portal thrombosis after lusutrombopag administration. Patients who achieved PLT counts of > 50,000 PLT µL^− 1^ had higher PLT counts before lusutrombopag administration. The degree of splenomegaly did not affect the rate of PLT count elevation. There was no specific adverse effect by administrating lusutrombopag for patients with PLT counts of around 50,000 µL^− 1^ but > 50,000 µL^− 1^.

**Conclusions:**

Lusutrombopag administration before RFA was effective and seemed to be relatively safe for hepatocellular carcinoma patients with low PLT counts.

**Trial registration:**

This study was approved by Japanese Red Cross Medical Center Institutional Reseach Comittie (#862, 07/03/2016), and was registered in a publically accessible primary register (#UMIN000046629, registered date: 14/01/2022).

## Introduction

Hepatocellular carcinoma (HCC) is one of the leading causes of death in Japan, and its treatment is one of the most important issues for hepatic medical professionals. Radiofrequency ablation is widely used in many countries, including Japan, as a local therapy aimed at eradicating neoplastic lesions that meet certain conditions in the liver, including hepatocellular carcinoma, and is a relatively minimally invasive treatment. However, it is classified as an invasive treatment, in that it involves puncturing the liver.

Hepatocellular carcinoma, on the other hand, often occurs in the background of cirrhosis of the liver, and patients with cirrhosis often show low platelet (PLT) counts; therefore, invasive procedure in such cases should always evaluate the risk of hemorrhage [1, 2]. Low platelet count was significant risk factors for hemorrhagic complication after RFA [1].

In recent years, lusutrombopag, an agonist of the thrombopoietin (TPO) receptor, has become available for use in the intensive open treatment of chronic liver disease patients complicated with low PLT counts [3–6]. Lusutrombopag acts selectively on human TPO receptors and promotes cell proliferation and culture induction from human bone marrow progenitor cells to megakaryocytes by activating the JAK-STAT and MAPK systems, which are part of the TPO signaling pathway, thereby increasing PLT count. Reported adverse effect of lusutrombopag are as follows: Thrombosis (1.3%): portal vein thrombosis, mesenteric vein thrombosis, etc. Skin: (< 2%): Rash. Blood: (less than 2%) decreased white blood cell count, (frequency unknown) decreased blood fibrinogen, increased fibrin D-dimer, increased FDP. Liver: (frequency unknown) increased AST, increased ALT, increased bilirubin. Gastrointestinal: (2–5%) Nausea. Neuropsychiatric system: (less than 2%) Headache. In patients with severe hepatic impairment, lusutrombopag should not be administered because it may increase blood levels.

We report our experience of using lusutrombopag in invasive procedures such as radiofrequency ablation (RFA) in our hospital and related institutions.

## Patients and methods

In this study, we conducted prospective observational study at our hospital and related facilities to examine safety and efficacy of lusutrombopag administered for HCC patients with low platelet count.

The enrollment criteria were as follows: Patients undergoing RFA for hepatocellular carcinoma with platelet counts below 50,000µL^− 1^ who received lusutrombopag. However, patients with platelet counts above 50,000µL^− 1^ but with a high risk of platelet counts below 50,000µL^− 1^ at the time of RFA were also enrolled and received lusutrombopag. Exclusion criteria were patients who were not eligible for RFA or who could not receive lusutrombopag. Not indicated for lusutrombopag were patients with allergic reactions to lusutrombopag or patients with a history of thrombosis. Written consent was obtained from all patients enrolled in the study.

Lusutrombopag 3 mg was administered once a day for 7 days to patients with low PLT levels (≤ 50,000 PLT µL^− 1^) scheduled for RFA. We have also administered lusutrombopag in some patients whose platelet counts exceed 50,000 µL^− 1^. Specifically, (1) cases in which the platelet count tends to decline and is between 50,000 and 60,000 (2) cases in which the platelet count has been below 50,000 in the past and is fluctuating around the 50,000 range. Lusutrombopag administration was commenced 6–27 days before implementing the invasive procedure. In general, lusutrombopag is usually administered 9–14 days before the procedure. However, due to problems with drug compliance by patients, emergency procedures, and the postponement of procedures due to any reason, there was some variation in the period from the start of lusutrombopag administration to the procedure. The following data of the patients was corrected regarding sex, age, background liver disease, platelet count, serum albumin level, selum total bililubin lever, prothrombin time, ascites, hepatic encephalopathy, spleen index (SI) mesured with ultrasonography, tumor size, number of tumor, and days between lusutrombopag administration and RFA procedure. Tumor staging was performed using the TNM classification of the Liver Cancer Study Group of Japan [7] and Barcerona-Clinic Liver Cancer (BCLC) staging [8]. Platelet transfusions were given to patients whose platelet levels were less than 50,000 immediately prior to RFA (0–2 days before RFA procedure).

The primary endpoint was the PLT transfusion avoidance rate at the time of the procedure. The effective (PLT blood transfusion avoidance) and the ineffective outcomes (PLT blood transfusion) were compared, and the predictors of the effective outcome were verified.

RFA was performed using the VIVA RF system (STARmed, Gyeonggi-go, Korea). The electrode was a 17-gauge, 20 cm long single needle of the same system. Percutaneous puncture was performed under echo guidance. The cauterization range of the electrode needle was 2 or 3 cm, and the 2 cm needle was started at 40 watts (W) and the 3 cm needle at 60 W. The power was increased by 20 W per minute and procedure was terminated after twe rolled offs, 6 min (2 cm needle) or 12 min (3 cm needle). In principle, needle tract ablation was not performed, but when bleeding from the needle tract was suspected by B-mode or Doppler ultrasonography, needle tract ablation was performed.

Statistical analysis was performed using a t-test for numerical variables and chi-square test or Fisher’s exact test for categorical variables. Pre- and Post- PLT count after administration of lusutrombopag was compared using paired t-test. Categorical variables were described as number (percentage) and numerical variables as median (range).

All procedures performed in studies involving human participants were in accordance with the ethical standards of the institutional research committee (#862, date of the first registration was 07/03/2016) and with the 1964 Helsinki declaration and its later amendments or comparable ethical standards. The experimental protocol was approved by Japanese Red Cross Medical Center institutional committee. All studies were performed in accordance with relevant guidelines and regulations. This study is registered in a publically accessible primary register (#UMIN000046629, registered date: 14/01/2022).

## Results

Lusutrombopag was administered to 91 patients before RFA from April 2016 to December 2019. The cohort included 33 females and 58 males with a median age of 71 years (range 51–86). The background liver disease was HCV in 42 cases, alcoholic liver disease (ALD) in 21 cases, HBV in 12 cases, NASH in 11 cases, and five others. The median Child-Pugh score was 7 (range: 5–11) points, with 44 cases of Child A, 44 cases of Child B, and three cases of Child C. The stage of HCC was Stage I in 37 cases, Stage II in 41 cases, Stage III in 12 cases, and Stage IV in one case (Table 1). Other background status of enrolled patients are shown in Table 1.

**Table 1:** Background of patients n = 91

Male/Female	58 (64) / 33 (36)
Age	71 (51–86)
Background liver disease	HBV 12 (13)
	HCV 42 (46)
	ALD 21 (23)
	NASH 11 (12)
	Others 5 (5)
Serum alubumin (g/dL)	3.7 (2.4–4.4)
Serum total bililubin (mg/dL)	1.3 (0.5–4.7)
Platelet count	4.6 (0.9–6.8)
Number of patients PLT > 50,000	29 (32)
Ascites +/-	73 (80) / 18 (20)
Encephalopathy +/-	84 (92) / 7 (8)
Child Pugh Score / Class	7 (5–11)
	A 44 (48)
	B 44 (48)
	C 3 (3)
ALBI score	-2.18 (-2.99 - -1.17)
mALBI grade	1 19 (21)
	2a 23 (25)
	2b 45 (50)
	3 4 (4)
Performance status 0 / 1	80 (88) / 11 (12)
Spleen Index	> 30 69 (76)
	20 ≦ SI ≦ 30 15 (16)
	<20 7 (8)
Tumor size (mm)	17 (8–40)
Number of tumor	1 (1–7)
TNM stage by LCSGJ	I 37 (41)
	II 41 (45)
	III 12 (13)
	IV 1 (1)
BCLC stage	0 15 (17)
	A 55 (60)
	B 8 (9)
	C 11 (12)
	D 2 (2)
Interval days*	13 (6–19)

*Days between lusutrombopag administration and RFA procedure

Categorical variables are shown as number (percentage) and numerical variables as median (Range)

**Fig. 1:**
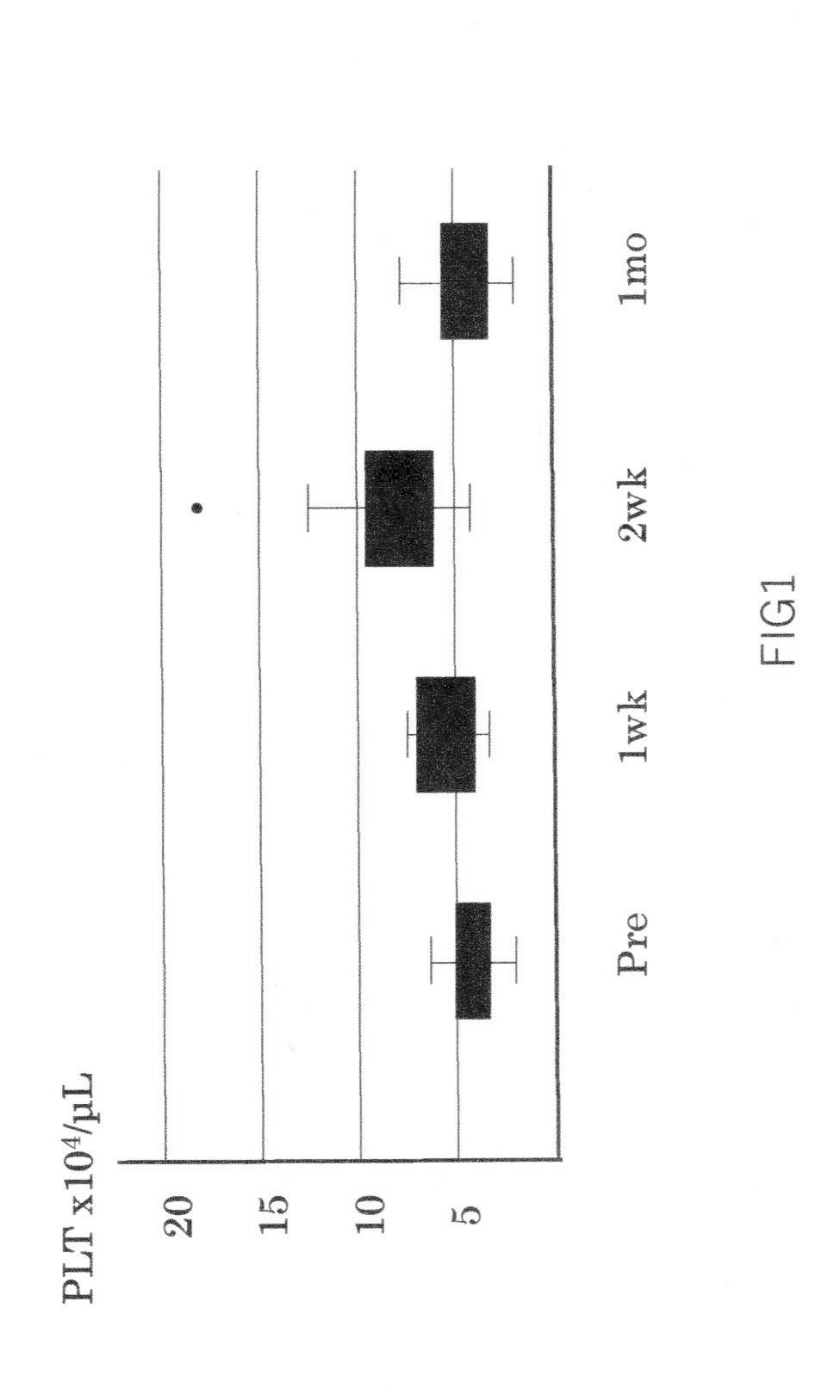
PLT count after administration of lusutrombopag PLT levels are shown before, 1 week, 2 weeks and 4 weeks after the start of treatment, with peak values at 9 to 14 days, and the procedure should be scheduled to allow for that period. Once elevated, the PLT level decreases to the pre-dose level after 4 weeks

The PLT level increased from 4.4 × 10^4^ ± 1.4 × 10^4^ to 8.6 × 10^4^ ± 2.5 × 10^4^ µL^− 1^ after administration of lusutrombopag (p < 0.0001) (Fig. 1). The number of days until the PLT counts showed their peak value was a median of 13 days (range 6–19 days). Among the 91 patients, 84 (92%) had PLT counts ≥ 50,000 µL^− 1^ before the procedure and were able to avoid PLT transfusion, but the remaining seven required PLT transfusions before the procedure. The rate of avoidance of PLT transfusion was 90% (6/62), even for patients with platelet levels less than 50,000 prior to lusutrombopag administration.There were no cases of hemorrhagic complications after the procedure, including those requiring PLT transfusion. Portal vein thrombosis was detected in one case (1.1%) among the RFA cases, but the elimination of the thrombus was observed after warfarin administration. Other adverse effect than portal vein thrombosis such as liver dysfunction or skin rush was not observed among patients after administration of lusutrombopag.

PLT count elevations of > 20,000 µL^− 1^ after lusutrombopag treatment were seen in 81/91 patients. The rate of PLT increase, defined as the (peak value − previous value)/previous value, was 108 ± 93%.

If patients who avoided PLT transfusion were considered effective cases, PLT counts were significantly higher in the effective cases than in the non-effective cases. Other factors such as sex, age, Child-Pugh Class, days to peak PLT count, and spleen index (SI) did not affect the efficacy of lusutrombopag (Table 2). There was no statistically significant difference between effective and non-effective groups regarding sex, age, Child-Class, days to peak PLT value, and spleen index, even for patients with platelet levels less than 50,000 prior to lusutrombopag administration.

**Table 2:** Comparison between effective and non-effective cases

	Effective	Non-effective	p value
n	84	7	
Sex Male(%)	53 (63)	5(71)	> 0.999
Age median(range)	71(51–86)	69(54–75)	0.702
Child Class A/ B + C	39/45	5/2	0.256
PLT pre (×10^4^ PLT µL^− 1^)	4.5 ± 1.1	3.4 ± 1.4	0.012
Number of patietns PLT > 50,000	28 (33)	1 (14)	0.4236
Days to peak PLT value	12(5–18)	14(6–19)	0.191
Spleen index 30> 30 ≦	20 64	2 5	0.674

P < 0.05: statistically significant

Categorical variables were described as number (percentage) and numerical variables as median (range). T-test for numerical variables and chi-square test or Fisher’s exact test for categorical variables

There was no difference in the rate of increase in PLT levels depending on the degree of splenomegaly before administration of lusutrombopag (Table 3).

**Table 3:** The rate of PLT elevation is dependant on spleen index

Total		108% ± 93%
SI	> 31	106% ± 60%
	21 < SI < 30	78% ± 35%
	< 20	75% ± 36%

The rate of PLT elevation (%) = (Peak-Pre)/Pre

**Fig. 2:**
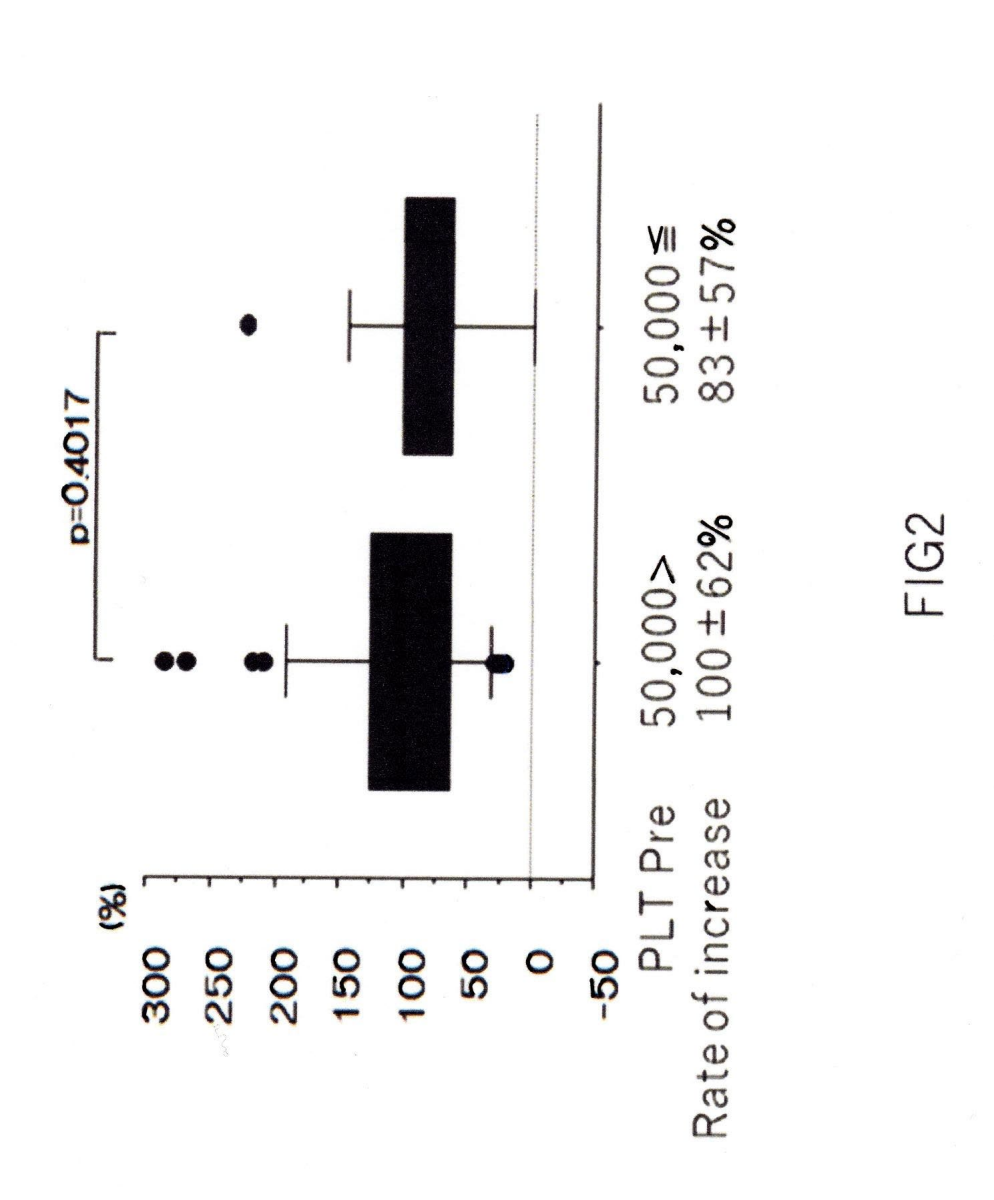
PLT elevation according to the PLT level before lusutrombopag administration PLT elevation according to the PLT level before lusutrombopag administration We examined whether there was a difference in the rate of PLT elevation depending on the pre-dose PLT level. There was no difference in the rate of PLT elevation between patients who met with a pre-dose PLT level of ≥ 50,000 PLT µL^− 1^ and those who met with a pre-dose PLT level of < 50,000 PLT µL^− 1^ (p = 0.4017)

Multiple administrations were performed twice in 10 cases, three times in three cases, five times in one case, and seven times in one case; no phenomenon in which the efficacy was diminished by repeated administration was observed. Patients treated seven times underwent RFA seven times in 2 years and 3 months for HBV-positive hepatocellular carcinoma, and each treatment could be carried out safely by raising PLT levels with lusutrombopag each time. Although we administered lusutrombopag to 29 patients with PLT counts > 50,000, there were no adverse effects caused by thrombocytosis. The rate of PLT elevation did not show a significant difference between patients with PLT counts > 50,000 and < 50,000 (Fig. 2).

## Discussion

In this study, by administrating lusutrombopag, 92% of patients with chronic liver disease and low PLT counts who underwent RFA for hepatocellular carcinoma avoided preoperative PLT transfusions. PLT transfusion should be avoided as much as possible because of the risk of unknown infections, use of limited medical resources, allergic reactions, and production of anti-PLT antibodies. We found that lusutrombopag could solve these problems. In addition, hepatocellular carcinoma treatment is often repeated due to the condition’s recurrence, and it is necessary to ensure a certain PLT count each time. Repeated PLT transfusions further increase the risk of anti-PLT antibody production. The safety and efficacy of lusutrombopag have been reported to be maintained even with repeated administration [9]. In our study, 15 patients received repeated administration of lusutrombopag, and all patients safely underwent RFA. Lusutrombopag did not diminish the efficacy of multiple doses, suggesting that repeated treatment could be safely performed with its administration.

There were no severe adverse reactions to lusutrombopag; portal vein thrombosis was observed in one patient, but the thrombus resolved after warfarin administration. According to previous reports, other mild side effects such as gastrointestinal disturbances and fever/malaise may be observed. However, the relationship with lusutrombopag administration is not clear, the incidence is low, and no severe side effects leading to drug discontinuation have been observed. As described above, the administration of lusutrombopag is relatively safe.

In a study of predictors of efficacy comparing effective and ineffective cases, the PLT count before lusutrombopag administration was significantly higher in effective cases. However, this result is not surprising, considering that effective cases were defined as those with PLT counts of ≥ 50,000. Therefore, we investigated whether there was a difference in the rate of PLT increase depending on the PLT count before administration, but no significant difference was observed between cases with a previous value of < 50,000 and those with a previous value of ≥ 50,000 (Fig. 2). In addition, some studies [10, 11] have reported a correlation between spleen size (degree of splenomegaly) and the efficacy of lusutrombopag, but this remains controversial. In this study, we used the rate of PLT increase as an evaluation criterion for comparison and found no correlation between the degree of splenomegaly and the rate of PLT increase after administration of lusutrombopag (Table 3).

Other treatment options for low PLT counts in patients with cirrhosis include splenectomy and splenic artery embolization, but both of these procedures are invasive and may be difficult to perform in elderly patients or those with reduced liver reserves. In this regard, the administration of lusutrombopag is minimally invasive and can achieve a high rate of PLT elevation, making it an effective treatment for patients with chronic liver disease who undergo invasive procedures.

Seven cases of lusutrombopag ineffectiveness were observed in our study, but in all cases platelet levels increased with platelet transfusion and RFA could be safely performed. In cases in which platelet transfusion is not successful, the other options described above may need to be considered.

Limitations of this study was that this is single arm obsevational study. At this point to set a control arm is difficult because of safety and efficacy of lusutrombopag.

Another limitation is the small number of patients enrolled. We have yet to obtain solid evidence of the safety and efficacy of rastrombopag. The discussion of predictors of efficacy is also not clear without a larger number of patients.

In conclusion, the administration of lusutrombopag before RFA and other invasive procedures seemed to be relatively safe, and PLT transfusion was avoided in more than 90% of patients. In patients with inadequate PLT elevation, PLT transfusion could be safely administered. Lusutrombopag may be useful as an alternative to transfusion in patients with low PLT levels during invasive procedures in chronic liver disease.

## Data Availability

The datasets used and/or analysed during the current study are available from the corresponding author on reasonable request.

## Abbreviations

PLT: Platelet
RFA: Radio Frequency Ablation
HCC: Hepatocellular Carcinoma
HCV: Hepatitis C virus
HBV: Hepatitis B virus
TPO: Thrombopoietin
